# A Fatal Consequence: Amiodarone-Induced Multiorgan Toxicity

**DOI:** 10.7759/cureus.62260

**Published:** 2024-06-12

**Authors:** Mohammed Al-Hiari, Ma’in Abumuhfouz, Leen Kayali, Utsab Panta, Carlos Rueda Rios

**Affiliations:** 1 Internal Medicine, Marshall University Joan C. Edwards School of Medicine, Huntington, USA; 2 Internal Medicine, University of Missouri Kansas City, Kansas City, USA; 3 Cardiovascular Disease, Marshall University Joan C. Edwards School of Medicine, Huntington, USA

**Keywords:** aipt (amiodarone-induced pulmonary toxicity), amiodarone-induced thyrotoxicosis, amiodarone-induced hepatotoxicity, multiorgan toxicity, amiodarone

## Abstract

Amiodarone is commonly used nowadays for the treatment of atrial fibrillation (AF). The wide use of this medication has led to the occurrence of adverse events, including pulmonary toxicity, hepatotoxicity, thyroid dysfunction, and many others. Higher doses of Amiodarone of ≥400 mg/day have been linked to increased complications.

We present a case of a 70-year-old male with multivessel coronary artery disease (CAD) with ischemic cardiomyopathy and severe peripheral artery disease (PAD) who underwent an elective left femoral to posterior tibial bypass surgery followed by percutaneous coronary intervention (PCI) complicated by new-onset AF. The patient was loaded with 150 mg of intravenous (IV) Amiodarone followed by 360 mg infusion over six hours for chemical cardioversion. The patient was then maintained on oral Amiodarone 400 mg/day until the day of presentation when he complained of progressive dyspnea. Imaging was significant for diffuse ground glass opacities and interstitial thickening. The echocardiogram revealed an improved ejection fraction (EF) of 40% from 20%. The patient had worsening oxygenation despite adequate IV diuresis and developed severe acute respiratory distress syndrome (ARDS) requiring mechanical ventilation (MV). A bronchoscopy with bronchoalveolar lavage (BAL) showed diffuse alveolar hemorrhage (DAH) with a high lymphocyte count and negative infectious disease testing. Lab tests revealed elevated liver enzyme levels. There were also changes in thyroid function from baseline with elevated free T4 at 1.83 ng/dL (0.8-1.4 ng/dL), suppressed thyroid stimulating hormone (TSH) at 0.109 mIU/mL (0.4-4 mIU/mL), negative anti-thyroglobulin (TG) antibodies, and anti-thyroid peroxidase (TPO) antibodies indicating a type 2 Amiodarone-induced thyrotoxicosis. Unfortunately, the patient’s condition deteriorated further despite appropriate treatment, and it was ultimately followed by his demise.

Severe, fatal cases of Amiodarone toxicity are scarce, but more reports are being seen. We strongly believe clinicians should have a high index of suspicion for Amiodarone-related adverse events in elderly males with cardiopulmonary comorbidities. It is imperative to have an increased understanding, greater vigilance, and closer monitoring of pulmonary function tests (PFTs), laboratory tests, and imaging studies.

## Introduction

Atrial fibrillation (AF) is widely considered the most prevalent electrophysiological disorder in clinical practice; almost one in 100 people worldwide suffer from this disease [1]. Many drugs have been introduced in the treatment of AF with Amiodarone being one of the most common and potent antiarrhythmic drugs. This class III antiarrhythmic drug has unique pharmacologic properties that allow it to treat all types of supraventricular and ventricular tachyarrhythmias, making it a very reliable medication. It has also been used for the prevention of AF perioperatively in thoracic surgeries [2].

Despite this medication being efficacious and having a low proarrhythmic potential, it is known to have notorious adverse effects in patients taking Amiodarone for a long period. Amiodarone has a long half-life of up to 100 days due to its lipophilic properties and a large volume of distribution, endorsing its accumulation in the body and toxicity. It is widely known that Amiodarone causes an adverse reaction in every organ, most notably the pulmonary, cardiac, gastrointestinal, hepatic, renal, neurologic, cutaneous, ocular, and thyroid systems. The severity of these effects can range from trivial ones that do not necessitate cessation of the therapy to serious ones that could prompt its discontinuation as some effects can be fatal [3].

Knowing the potential toxicities and complications of Amiodarone is essential in clinical practice, and a close follow-up is of great importance. It is not uncommon to manage cases of systemic toxicity due to Amiodarone; however, to our knowledge, it is unusual to see patients with more than two organ involvements. We noted a subacute Amiodarone-induced multiorgan toxicity leading to a fatality within five months after initiation of the treatment.

## Case presentation

**Figure 1:**
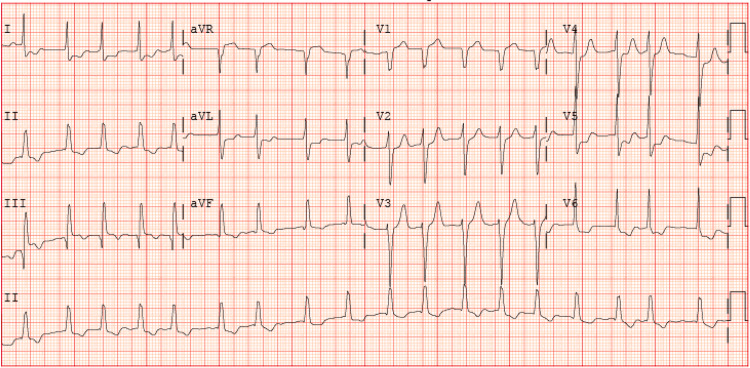
EKG showing a new-onset AF with RVR EKG, electrocardiogram; AF, atrial fibrillation; RVR, rapid ventricular response

**Figure 2:**
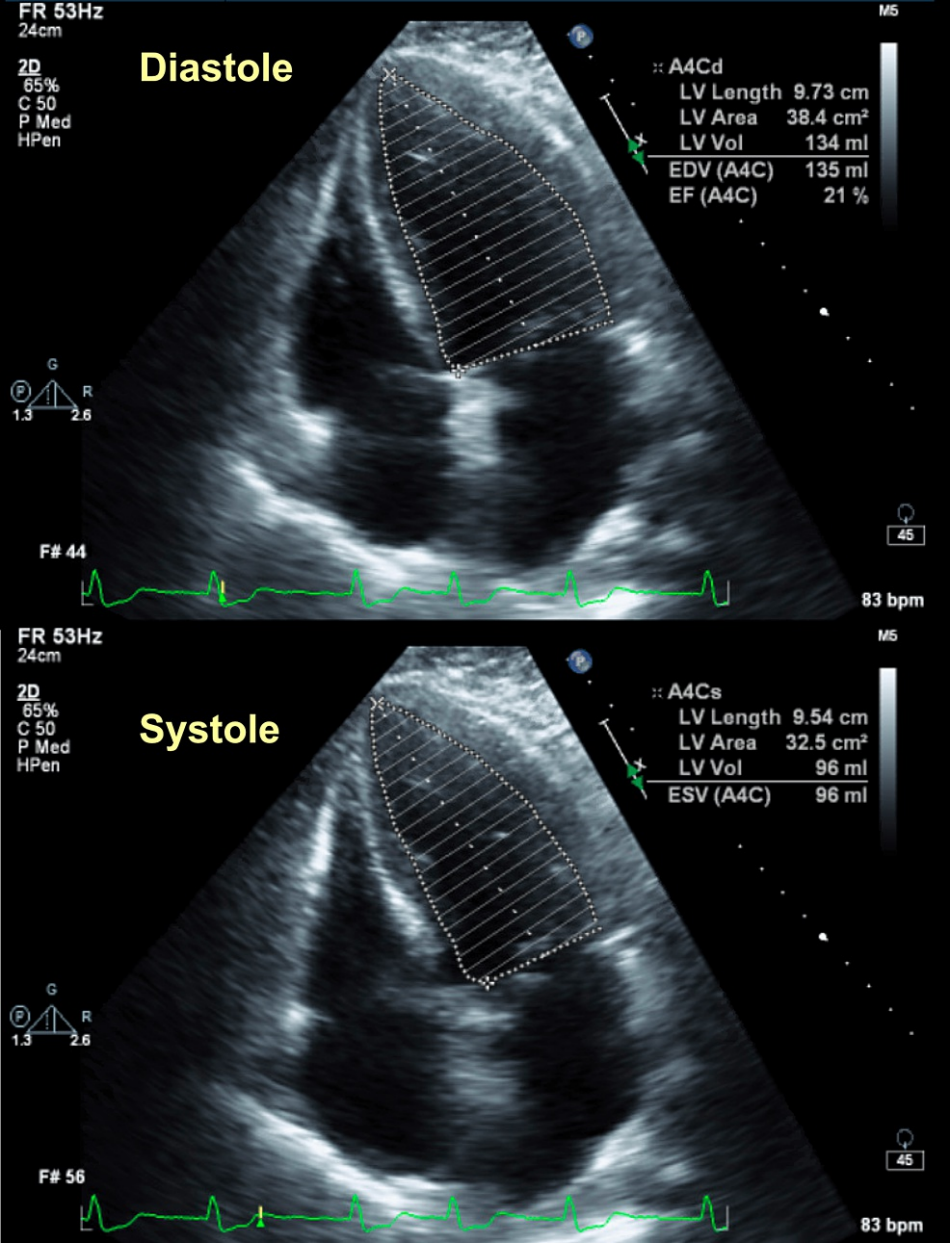
A TTE of the patient during his first admission showing evidence of acute HF with an estimated EF of 15-20%. The EF was measured by a cardiologist and the numbers shown here were inserted by the technician before being reviewed by the cardiologist TTE, transthoracic echocardiogram; HF, heart failure; EF, ejection fraction

The patient was a 70-year-old male with a past medical history of coronary artery disease (CAD) requiring percutaneous coronary intervention (PCI) and stenting of the first branch of the obtuse marginal (OM1), severe peripheral arterial disease, metabolic syndrome, and no known respiratory disease other than obstructive sleep apnea using a continuous positive airway pressure (CPAP) machine at night consistently. The patient was undergoing an elective left femoral to posterior tibial bypass surgery that was well tolerated with no intraoperative complications. He developed a new onset of AF, as seen in the patient’s electrocardiogram (EKG) (Figure 1), along with an elevation of the troponin-I levels up to 12,760 pg/mL (3-79 pg/mL). An urgent transthoracic echocardiogram (TTE) revealed hypokinesis of the entire inferior wall along with a severely reduced left ventricular systolic function and an ejection fraction (EF) of 15-20% (Figure 2) prompting an urgent transfer to a nearby PCI capable facility after initiating dobutamine infusion given the patient was in shock.

The patient underwent an urgent left heart catheterization (LHC) showing evidence of multivessel CAD with chronic total occlusion (CTO) of the OM1 (in-stent restenosis), 50% occlusion of the first diagonal branch (D1), 80% occlusion of the second diagonal branch (D2), 90% stenosis of the mid-portion of the left anterior descending artery, and CTO of the mid-portion of the right coronary artery. EF was measured to be 20% on left ventriculography. After consulting the cardiothoracic surgeons, it was determined the patient would benefit from PCI rather than bypass, and it was performed successfully to the mid-portion of the LAD and D2. His AF was controlled after chemical cardioversion was initiated with intravenous (IV) Amiodarone 150 mg followed by a continuous infusion of 360 mg/200 mL at a rate of 33 mL/hour for six hours total before transitioning to an oral form of Amiodarone 200 mg twice daily for maintenance. His baseline lab work (Table 1) at that hospital stay showed normal levels of liver function test (LFT) and creatinine clearance, and a baseline pulmonary function test (PFT) (Table 2) was obtained showing a suspected, mild restrictive pattern, although no lung volume measurements were performed. Dobutamine infusion was then weaned off, the patient was started on guideline-directed medical therapy for CAD and heart failure (HF), and he was then discharged to an inpatient rehabilitation facility with a wearable cardioverter defibrillator.

**Table 1:** Comprehensive metabolic panel BUN, blood urea nitrogen; eGFR, estimated glomerular filtration rate; Na+, sodium; K+, potassium, Cl-, chloride; HCO_3_, bicarbonate; AGAP, anion gap; Ca^+2^, calcium; Mg^+2^, magnesium, PO^-4^, phosphorus; ALP, alkaline phosphatase; AST, aspartate aminotransferase; ALT, alanine aminotransferase; SGOT, serum glutamic-oxaloacetic transaminase; SGPT, serum glutamic-pyruvic transaminase; PT, prothrombin time; INR, international normalized ratio; LDH, lactate dehydrogenase

Comprehensive metabolic panel					
	Baseline	Admission	During	Reference	Units
Glucose	153	279	303	70-100	mg/dL
BUN	15	29	94	7-27	mg/dL
Creatinine	1.0	1.3	1.6	0.6-1.3	mg/dL
eGFR	74	54	43	>90	ml/min/1.73m^2^
Na^+^	143	130	157	135-145	mmol/L
K^+^	4.1	4.3	3.4	3.3-5.1	mmol/L
Cl^-^	103	100	116	96-108	mmol/L
HCO_3_	33	27	37	21-32	mmol/L
AGAP	7	3	3	4-16	mmol/L
Ca^+2^	8.4	8.4	8.3	8.5-10.1	mg/dL
Mg^+2^	2.3	1.9	2.7	1.7-2.4	mg/dL
PO^-4^	2.9	3.4	4.1	2.5-4.9	mg/dL
Albumin	2.4	2.7	2.9	2.9-4.9	g/dL
Total protein	5.6	5.7	5.5	6.4-8.2	g/dL
Total bilirubin	1.1	1.3	3.3	0.1-1.0	mg/dL
Direct bilirubin	0.4	0.4	Not present	0.1-0.3	mg/dL
ALP	62	97	129	50-120	U/L
ALT/SGPT	20	56	89	15-65	U/L
AST/SGOT	14	84	58	5-37	U/L
PT	13.4	13.6	22.2	10.7-13.5	Sec
INR	1.1	1.3	1.8	0.8-1.1	N/A
LDH	Not present	471	683	94-250	U/L
Lactic acid	1.5	2.1	2.3	0.5-2.0	mmol/L

**Table 2:** Baseline pulmonary function test LLN, lower limit of normal; FVC, forced vital capacity; FEV, forced expiratory volume; FIVC, forced inspiratory vital capacity; FIF max, maximal forced inspiratory flow; MVV, maximal voluntary ventilation; PFT, pulmonary function test

Spirometry	Actual	Pred	Pred%	LLN	Units
FVC	1.92	4.08	47	3.21	L
FEV1	1.50	2.99	50	2.25	L
FEV1/FVC	78	74	105	64	%
FEV6	1.92	3.84	50	2.98	L
FEV6/FVC	100	94	106	N/A	%
FEV 25%	3.51	7.62	46	5.03	L/sec
FEV 75%	0.46	1.29	35	0.12	L/sec
FEV 25-75%	1.33	2.27	58	0.75	L/sec
FEV max	3.97	7.87	50	5.68	L/sec
FIVC	1.93	N/A	N/A	N/A	L
FIF max	2.37	N/A	N/A	N/A	L/sec
MVV	47	120	39	72	L/min

**Figure 3:**
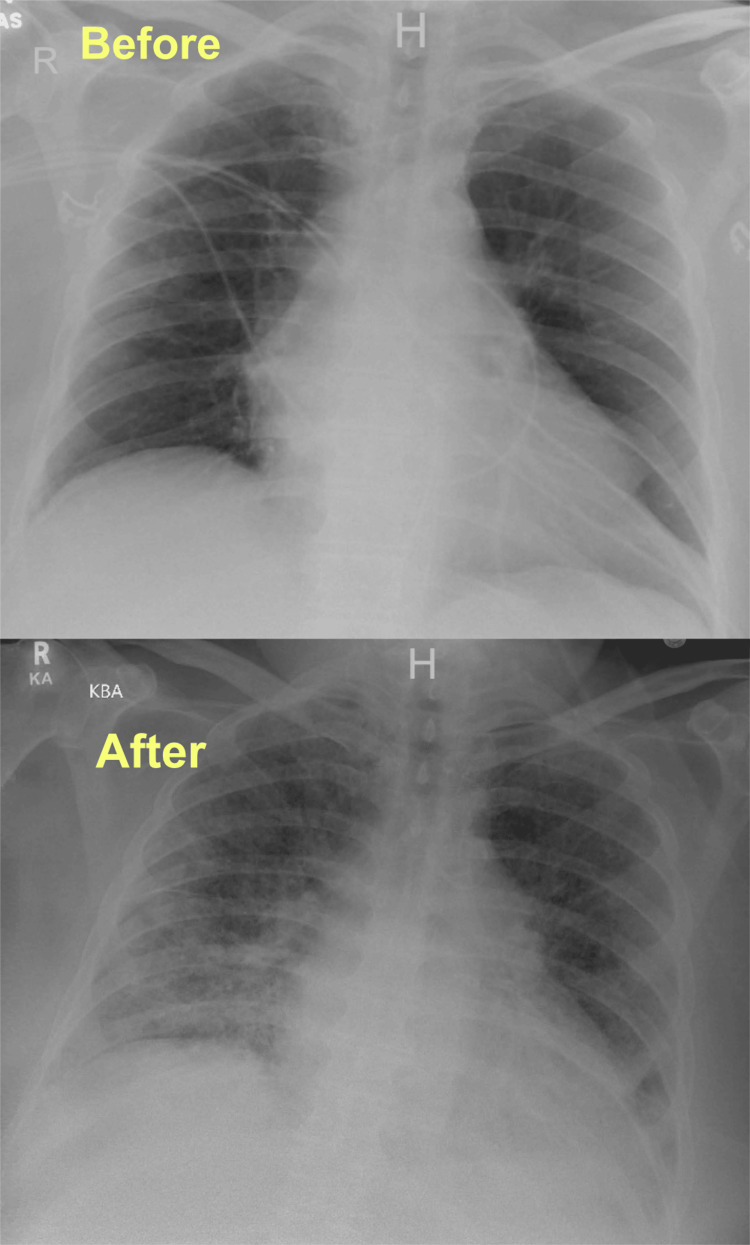
A picture of the patient’s baseline CXR (above) and his CXR at the second admission (below) revealing new onset diffuse bilateral opacities and airspace disease with blunting of the costophrenic angles CXR, chest x-ray

The patient was followed at an HF clinic afterward until five months after discharge when he presented with gradual worsening of shortness of breath on minimal exertion, orthopnea, and a productive cough. The patient’s SaO_2_ dropped to 76% while on room air, he was in respiratory distress, and using accessory muscles. The patient was placed accordingly on CPAP with an improvement in his saturation. Physical examination also revealed +1 pitting edema in both lower extremities and decreased bibasilar air entry with inspiratory crackles. Vitally, the patient’s blood pressure (BP) was within normal limits (WNL), his heart rate was controlled, and he was afebrile. Arterial blood gas (ABG) on admission showed significant hypoxemia (Table 3) and other lab work was concerning for lactic acidosis and acute kidney injury (Table 1). The pro-brain natriuretic peptide (pro-BNP) was 3,163 pg/mL (5-800 pg/mL), although lower than how it was on the last admission, and the troponin-I level was WNL (Table 4). Chest x-ray (CXR) on admission showed new onset diffuse bilateral opacities and airspace disease with blunting of the costophrenic angles (Figure 3).

**Table 3:** ABG PCO_2_, partial pressure of carbon dioxide; PO_2_, partial pressure of oxygen; HCO_3_, bicarbonate; FiO_2_, fraction of inspired oxygen; ABG, arterial blood gas

	Baseline	Admission	During	Reference	Units
pH	7.46	7.37	7.35	7.35-7.45	N/A
PCO_2_	35	44	70	35-45	mmHg
PO_2_	101	60	84	83-100	mmHg
HCO_3_	31	27	41	23-27	mmol/L
FiO_2_	28	32	100	N/A	%

**Table 4:** Cardiac labs BNP, brain natriuretic peptide

	Baseline	Admission	During	Reference	Units
NT-Pro BNP	4,275	3,163	20,510	5-800	pg/mL
Troponin-I high sensitivity	65	72	Not present	3-79	pg/mL

**Figure 4:**
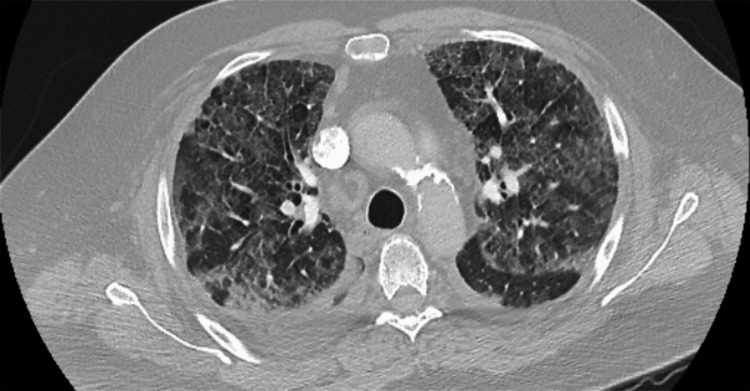
A CT of the chest showing diffuse interlobular septal thickening with superimposed patchy ground-glass opacities or “crazy-paving” pattern CT, computed tomography

**Figure 5:**
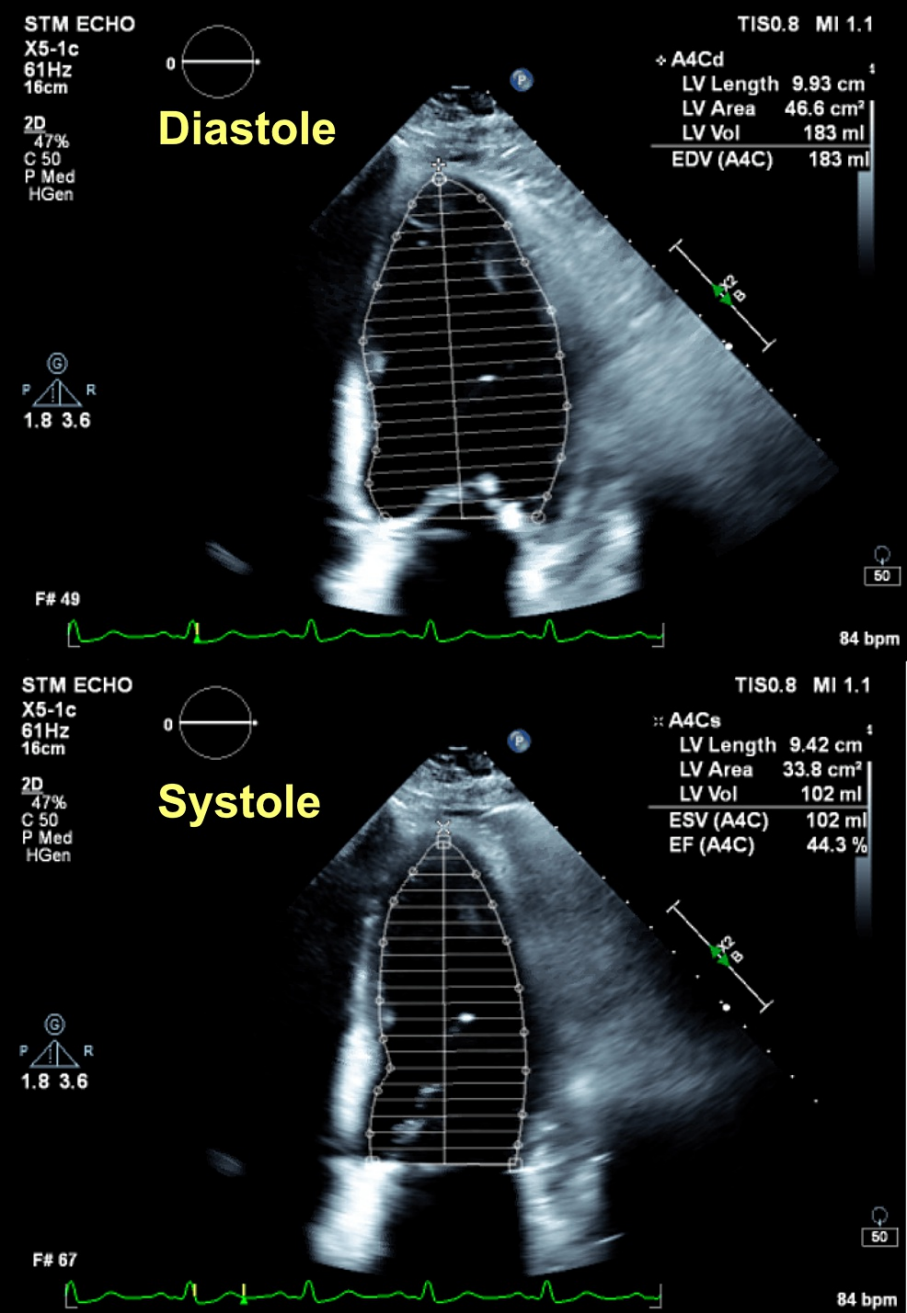
A TTE of the patient during his second admission showing evidence of an improved EF of up to 40%. The EF was measured by a cardiologist and the numbers shown here were inserted by the technician before being reviewed by the cardiologist TTE, transthoracic echocardiogram; EF, ejection fraction

Computed tomography (CT) of the chest with IV contrast pulmonary embolism (PE) protocol ruled out the possibility of having a PE; however, it was concerning for the presence of marked, diffuse, interlobular septal thickening with superimposed patchy ground-glass opacities, what is known as “crazy-paving” pattern, seen throughout both lung fields and small bilateral pleural effusion (Figure 4). An urgent TTE was performed that showed mild global hypokinesis with an improved EF of up to 40% from 20% previously (Figure 5), no significant valvular heart disease, and normal right ventricular (RV) systolic function, although the RV systolic pressure was increased up to 45 mmHg (less than 20 mmHg). The patient was maintained on a CPAP and was initiated on IV diuresis with Furosemide, Metolazone, and Spironolactone. The white blood cell count (WBC) was WNL (Table 5), the procalcitonin level was low (Table 6), two sets of blood cultures were negative for any growth, and atypical pneumonia workup, including urine Legionella antigen (Ag), urine *Streptococcus pneumoniae* Ag, and *Mycoplasma pneumoniae* DNA testing, were all negative. The patient subsequently was started on community-acquired pneumonia coverage with IV Ceftriaxone 1 g daily and Azithromycin 500 mg daily.

**Table 5:** Complete blood count WBC, white blood cells; RBC, red blood cells; Hgb, hemoglobin; Hct, hematocrit; MCV, mean corpuscular volume; MPV, mean platelet volume

	Baseline	Admission	During	Reference	Units
WBC	8.7	9.7	20.5	4.5-10.0	×10^^9^/L
RBC	4.37	4.02	2.47	4.4-5.9	×10^^12^/L
Hgb	11.7	10.9	6.8	14.0-16.0	g/dL
Hct	39.9	32.4	20.7	40.0-52.0	%
MCV	91.3	85.8	83.3	80.0-96.0	fL
Platelet	201	125	159	150-440	×10^^9^/L
MPV	13.0	12.4	12.3	9.0-12.0	fL

**Table 6:** Inflammatory markers ESR, erythrocyte sedimentation rate; CRP, C-reactive protein

	Baseline	Admission	During	Reference	Units
ESR	10	27	61	0-16	mm/HR
CRP	Not present	21.7	31.3	0.0-0.3	mg/dL
Procalcitonin	0.09	0.05	0.22	0.10-0.25	ng/dL

**Figure 6:**
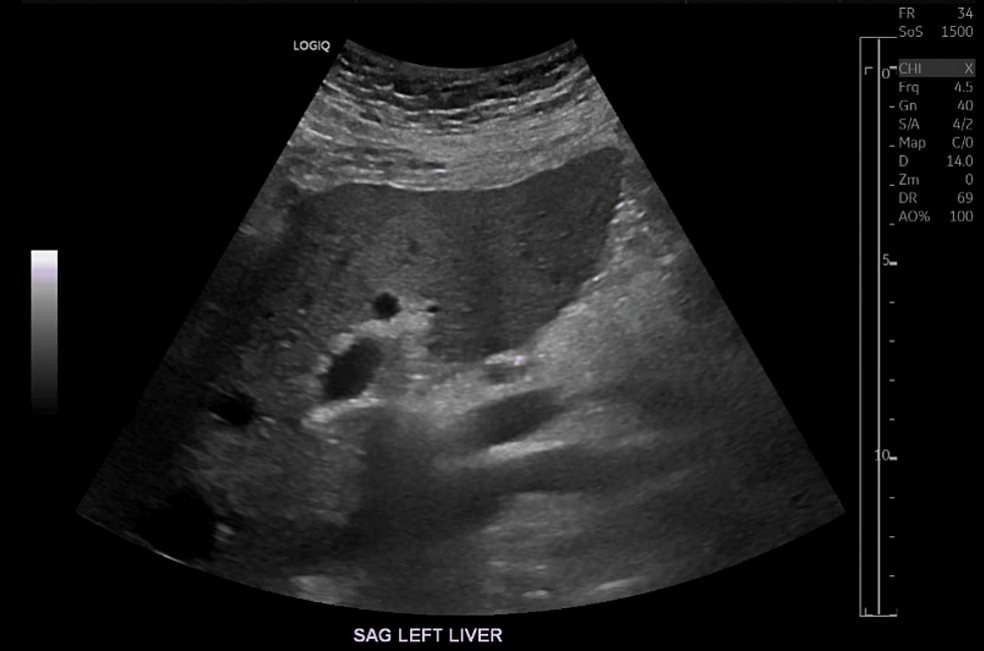
A sagittal cut of the liver US showing cirrhotic changes manifested by diffuse nodularity of the surface of the liver US, ultrasound

Further lab work revealed a new onset of transaminitis and direct hyperbilirubinemia (Table 1). A hepatitis panel was performed being merely reactive to hepatitis A antibodies and no evidence of active or chronic hepatitis infection. CT scan of the abdomen and pelvis was consistent with cirrhotic changes manifested by diffuse nodularity of the liver and splenomegaly (Figure 6); this was confirmed with a liver ultrasound (US). The patient is not known to have a history of alcohol consumption or high-risk medications for hepatotoxicity before the initiation of Amiodarone. Thyroid studies were also obtained showing a low level of thyroid-stimulating hormone (TSH), an elevated free T4 (FT4), and a low T3 level (Table 7). Thyroid gland US was consistent with a normal thyroid glandular size, no discrete nodularity, and Doppler imaging demonstrated no evidence of significant hyperemia. Anti-thyroglobulin (TG), anti-thyrotropin receptor, anti-thyroid stimulating immunoglobulins (TSI), and anti-thyroid peroxidase (TPO) antibodies were all absent. Subsequently, a decision to initiate glucocorticoid therapy was made, and he was started on IV Methylprednisolone 60 mg every eight hours.

**Table 7:** TFT FTI, free thyroxine index; TSH, thyroid stimulating hormone; TG, thyroglobulin; TPO, thyroid peroxidase; TSI, thyroid stimulating immunoglobulins; TR, TSH receptor; TFT, thyroid function test

	Result	Reference	Units
Total T4	11.9	4.5-12.5	ug/dL
Free T4	1.83	0.80-1.40	ng/dL
Free T3	1.07	1.80-4.20	pg/mL
T3 uptake	42	31-39	%
FTI	5.0	1.5-4.5	Units
TSH 3rd gen	0.109	0.400-4.000	mIU/mL
TG antibodies	<15.0	15.0-60.0	IU/mL
TPO antibodies	<28.0	28.0-60.0	IU/mL
TSI	<0.10	0.00-0.55	IU/L
TR antibodies	<1.10	0.00-1.75	IU/L

**Figure 7:**
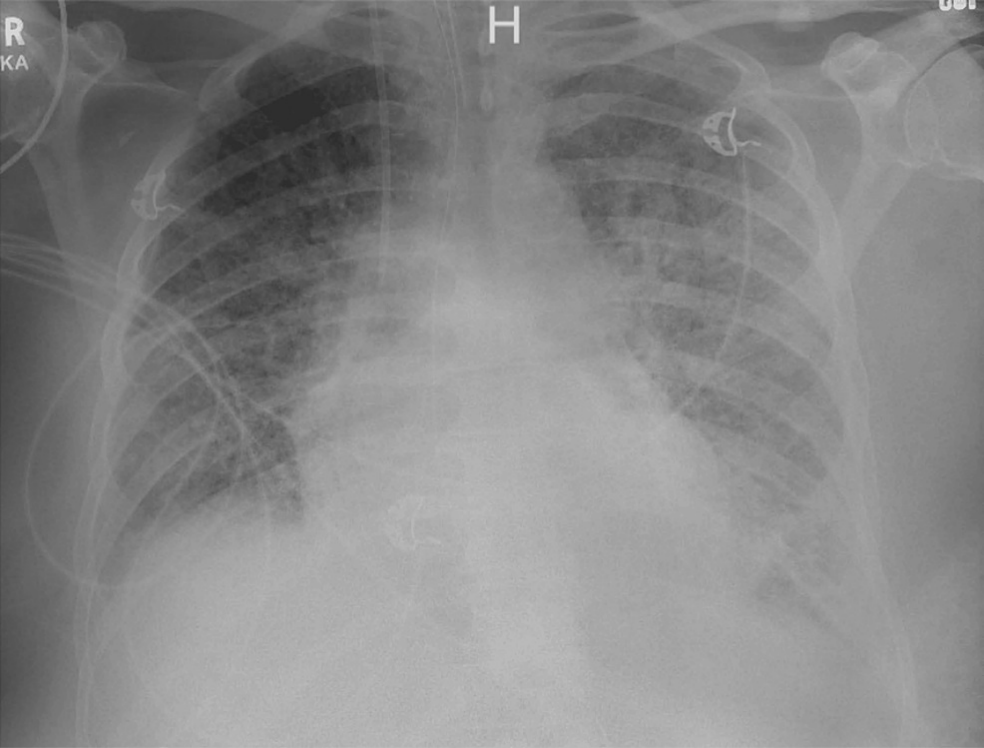
A repeat CXR showing worsening of the diffuse airspace disease bilaterally more on the left field with blunting of the costophrenic angle CXR, chest x-ray

The patient’s condition deteriorated further despite aggressive diuresis and optimal medical therapy; his oxygen requirements further increased, he was required to be on a non-invasive ventilator continuously, and he had an elevating pro-BNP level (Table 4). His hemoglobin level gradually dropped to less than 7 g/dL (14-16 g/dL) requiring a blood transfusion (Table 5). Eventually, the patient became distressed, tachypneic, and had impending respiratory failure prompting an intensive care unit transfer following endotracheal intubation with mechanical ventilation (MV). ABG post-intubation showed a PaO_2_/FiO_2_ ratio of less than 100 (Table 1) and a repeat chest imaging showed worsening of the airspace disease (Figure 7). The patient’s BP dropped significantly to a mean arterial pressure of less than 65 mmHg, lactic acid was elevated at 2.3 mmol/L (0.4-2 mmol/L), and his lactate dehydrogenase (LDH) level was 683 U/L (94-250 U/L), prompting Norepinephrine infusion initiation and escalating his antibiotic regimen for broader coverage with IV Vancomycin 1.5 g every 12 hours and IV Cefepime 1 g every 6sixhours.

A bronchoscopy was performed showing evidence of diffuse alveolar hemorrhage (DAH) with no active bleeding and a bronchoalveolar lavage (BAL) sample was obtained and sent for analysis, culture, and cytology. The results of which did show an elevated WBC level predominantly neutrophils along with a high level of RBC (Table 8). Infectious workup on the BAL sample, including cultures, Aspergillus Ag, galactomannan, and a viral polymerase chain reaction (PCR) panel, was negative for any pathogenic growth and showed no more than normal flora. His erythrocyte sedimentation rate (ESR) and C-reactive protein (CRP) were significantly elevated (Table 6), but despite that, a new set of blood and urine cultures did not show any growth. Autoimmune and vasculitic workups were then obtained including anti-glomerular basement membrane (GBM) antibodies, anti-myeloperoxidase (MPO) antibodies, and anti-nuclear antibodies (ANA) that were all absent (Table 9). Complement C3 level was WNL but C4 was mildly elevated.

**Table 8:** BAL WBC, white blood cell; RBC, red blood cells; BAL, bronchoalveolar lavage

	Result	Reference	Units
Color	Red	N/A	N/A
Turbidity	Cloudy	N/A	N/A
WBC	885	0-5	uL
RBC	108,148	Not present	uL
Neutrophil	90	Not present	%
Lymphocyte	4	0-5	%
Monocyte	2	Not present	%
Cytology	Benign bronchial epithelial cells, pulmonary macrophages, and acute Inflammation. No evidence of malignancy.		

**Table 9:** Serology C3, complement component 3; C4, complement component 4; GBM, glomerular basement membrane; MPO, myeloperoxidase; PR3, proteinase 3; ANA, antinuclear antibodies

	Result	Reference	Units
C3	146	90-180	mg/dL
C4	42	10-40	mg/dL
GBM antibodies	<0.2	0.0-0.9	Units
MPO antibodies	<0.2	0.0-0.9	Units
PR3 antibodies	<0.2	0.0-0.9	Units
ANA	<1:80 (negative)	<1:80	N/A

Despite the use of bronchodilators, steroids, and appropriate sedation and analgesia via Propofol, Midazolam, and Fentanyl infusion while on the MV, his respiratory status continued to be severely compromised to the point he was initiated on paralytics via Cistacurium infusion as a last resort. Along with the worsening of the patient’s labs, his outcomes seemed unfavorable despite maximum therapy, and the chances of a significant clinical improvement were slim. After a lengthy discussion with the patient’s family and healthcare proxy regarding the patient’s current situation and future outcomes, withdrawal of lifesaving measures by extubating the patient, withdrawing therapeutic medical treatment, and initiating comfort measures were deemed necessary. Not long was it before the patient expired and was pronounced dead.

## Discussion

Amiodarone is a widely prescribed medication with a high level of effectiveness, which makes it appealing to prescribe whether in the inpatient setting or outpatient [4]. Amiodarone primarily blocks the potassium rectifier currents ultimately prolonging the repolarization phase. Unlike other antiarrhythmic drugs, Amiodarone works through multiple pathways simultaneously; it blocks beta-adrenergic receptors, calcium channels, and sodium channels inhibiting sinoatrial node automaticity and atrioventricular nodal conduction velocity. Moreover, Amiodarone is highly lipophilic, which explains its long half-life; however, medication could pose a higher risk by causing adverse events, and, correspondingly, the pharmacological effect of this drug may last for up to three months after its discontinuation [5]. In our case, the high potency of this medication came at the expense of systemic toxicity in several body organs simultaneously.

The exact mechanism for Amiodarone toxicity is yet to be concluded; however, multiple theories may explain the phenomenon behind it with the most acceptable being the "cytotoxic" and the "immunological" theories imposed by the metabolites of Amiodarone and Desethylamiodarone. The risk of Amiodarone toxicity is particularly elevated in patients who consume 400 mg/day for more than two months or 200 mg/day for over two years, although it can occur in lower cumulative doses, perhaps there is indeed no actual "safe" dose for Amiodarone as highlighted by the author of this article [6,7].

The most serious Amiodarone-related adverse effect is lung toxicity. In addition to the previously discussed theory, it can also contribute to forming free radicals leading to cellular damage and programmed cell death. On a microscopic level, lung biopsies among patients with Amiodarone-induced pulmonary toxicity (AIPT) revealed lymphocytic infiltration, accumulation of lipid-laden "foamy" macrophages, and interstitial fibrosis. There are various patterns of lung involvement; chronic interstitial pneumonia, bronchiolitis, acute respiratory distress syndrome (ARDS), DAH, pleural effusion, and pulmonary nodules or masses. Corticosteroid therapy should be considered in patients showing extensive lung involvement on imaging or those who develop hypoxemia; a starting dose of 40 to 60 mg/day of Prednisolone is reasonable. Prognosis is favorable when the disease is detected early before it becomes advanced, while mortality is highest in patients who progress to ARDS, our patient included [6]. AIPT should be differentiated from HF; in this case, the EF improved between the two visits that he had; pro-BNP was lower than the prior admission, and his pulmonary status did not improve despite adequate diuresis, which helped us rule out cardiac causes for his deteriorating lung function.

Although uncommon, the possible reason for hepatotoxicity in our case is Amiodarone as the production of reactive oxygen species can lead to hepatic triglyceride accumulation and microvesicular steatosis in hepatocytes [8]. Amiodarone hepatotoxicity can occur in two clinical scenarios: during rapid IV administration and through prolonged oral consumption [9]. A case by Akbal et al. presented a patient who suffered from acute liver failure after short-term use of oral Amiodarone, which is quite uncommon but proves that even immediate use of oral Amiodarone can cause liver damage, similar to our patient, but it can be worse up to the point where the patient may develop cirrhosis and fulminant liver failure with the prolonged use of Amiodarone [10]. It is optimal to obtain a liver biopsy to identify patients at high risk for progression to cirrhosis if it was not for the instability of the patient’s hemodynamic status [9,10]. Approximately 25% of patients using Amiodarone develop a transient asymptomatic increase in serum transaminases that resolves spontaneously or after dose reduction [11]. In extensive patient studies of chronic amiodarone therapy, there has not been significant variation observed in other liver chemistry markers like alkaline phosphatase, gamma-glutamyl transferase, bilirubin, and LDH [9]. Amiodarone-induced symptomatic hepatitis, cirrhosis, and fatal hepatic failure are extremely rare (0-3%); however, when the diagnosis is established, the mortality risk may be as high as 60% at five months [11].

A similarity between Amiodarone and thyroid hormones exists due to its iodine content. Even low doses of Amiodarone have been linked to a fourfold increase in the likelihood of developing thyroid dysfunction, and lowering the maintenance dose has been found to have decreased the prevalence of these adverse effects [12]. The iodine excess mediated by giving Amiodarone is capable of causing both hyper- and hypothyroidism; in a state of thyroid deficiency, the excess iodine load would cause excessive production of thyroid hormones by binding to different glycoproteins in the thyroid glands. On the other hand, in a euthyroid state, the iodine excess can lead to an inhibitory effect described by Wolff-Chaikoff in 1963; it inhibits the production of iodine transporters ultimately leading to a decrease in the concentration of iodine in the cells and ultimately inhibits the production of thyroid hormones [13]. Another mechanism where thyroid dysfunction can occur secondary to Amiodarine is the direct cellular cytotoxicity to the follicular cells of the thyroid glands, and glucocorticoids may be employed to expedite the healing process. It is of great importance to restore the euthyroid state in such patients as thyrotoxicosis can worsen an already dire cardiac dysfunction, which would increase mortality [14].

## Conclusions

As demonstrated, Amiodarone can lead to various toxicities and side effects involving different organ systems; however, it is exceedingly rare, to our knowledge, to observe multiorgan toxicity simultaneously in a single patient. Our patient presented with a case of Amiodarone toxicity involving three different organs: the lungs, liver, and thyroid gland. It is imperative for patients receiving maintenance doses of Amiodarone to be frequently assessed for toxicity and obtain screening tests. The guidelines recommend a baseline PFT, CXR, thyroid function test (TFT), LFT, EKG, skin, and eye examination. Periodic monitoring of different tests is recommended, which can be every one to three months and some are semiannual or annual.

Unfortunately, our patient did not have a proper follow-up; the importance of adhering to follow-ups should be explained to all patients on Amiodarone as early detection of adverse effects and the prompt discontinuation of the culprit drug can be lifesaving for them. Furthermore, adopting the practice of using the minimal possible and effective dose in these patients can prevent the occurrence of systematic toxicity.
